# Role of Orexin-1 Receptor Within the Ventral Tegmental Area in Mediating Stress- and Morphine Priming-induced Reinstatement of Conditioned Place Preference in Rats

**DOI:** 10.32598/bcn.9.10.130

**Published:** 2019-07-01

**Authors:** Ronak Azizbeigi, Zahra Farzinpour, Abbas Haghparast

**Affiliations:** 1. Department of Physiology, Faculty of Veterinary Medicine, Sanandaj Branch, Islamic Azad University, Sanandaj, Iran.; 2. CAS Key Laboratory of Brain Function and Disease, School of Life Sciences, University of Science and Technology of China, Hefei, Anhui, China.; 3. Neuroscience Research Center, School of Medicine, Shahid Beheshti University of Medical Sciences, Tehran, Iran.

**Keywords:** Reward, Orexin system, Reinstatement, Ventral tegmental area, Forced swim stress, Conditioned place preference

## Abstract

**Introduction:**

Orexin-containing neurons exist in the lateral hypothalamic region, sending their projections toward mesolimbic regions such as the Ventral Tegmental Area (VTA).

**Methods:**

In the current study, a Reinstatement model is used to examine the effects of intra-VTA administration of SB334867 as an Orexin-1 Receptor (OX1R) antagonist on drug priming- and Forced Swim Stress (FSS)-induced reinstatement of morphine. Eighty-eight male adult albino Wistar rats, weighing 200–280 g, were bilaterally implanted by cannulas into the VTA. We induced the Conditioned Place Preference (CPP) by Subcutaneous (SC) injection of morphine (5 mg/kg) daily in three days. Then, the CPP score was calculated. After a 24-h “off” period following achievement of extinction criterion, the rats were tested for drug priming-induced reinstatement by a priming dose of morphine (1 mg/kg, SC) and for FSS-induced reinstatement 10 min after FSS. In the next experiments, the animals received different doses of intra-VTA administration of SB334867 (0.3, 3, and 1 nM/0.3 μL 12% DMSO per side) and bilaterally were subsequently tested for FSS- and morphine priming-induced reinstatement.

**Results:**

Our findings indicated that the FSS could induce the reinstatement of seeking behaviors. Furthermore, intra-VTA administration of OX1R antagonists suppressed FSS- and drug priming-induced reinstatement dose-dependently.

**Conclusion:**

It is concluded that FSS and drug priming-induced reinstatement might be mediated, at least in part, by stimulation of orexin receptors in the VTA.

## Highlights

- Forced swim stress (FSS) induced the reinstatement of morphine seeking in rats.
- Orexin-1 receptor blockade in the intra-ventral tegmental area reduced morphine-induced reinstatement.
- Orexin-1 receptor blockade in the intra-ventral tegmental area reduced FSS-induced reinstatement.

## Plain Language Summary

We designed this study to investigate effects of intra-VTA (ventral tegmental area) administration of SB334867 as an orexin-1 receptor (OX1R) antagonist on drug priming- and forced swim stress (FSS)-induced reinstatement of morphine. The conditioned place preference (CPP) was induced by injecting morphine and the reinstatement by the administration of effective priming dose of morphine. The extinguished rats received an intra-VTA injection of SB334867 before effective priming dose injection of morphine. In others, the extinguished rats were given intra-VTA administration of SB334867 bilaterally and were subsequently tested for FSS- and morphine priming-induced reinstatement. Our results indicated that intra-VTA administration of SB334867 could inhibit morphine priming - and FSS-induced reinstatement of extinguished morphine seeking in the rats. It seems that OX1R in the VTA may be involved in the reward system and could play an important role in the effect of stress on reinstatement of morphine-seeking behaviors in this area.

## 1. Introduction

Drug addiction can be conceptualized as a recurrent disorder characterized by the high rate of relapse to drug taking following long periods of abstinence (Koob & Kreek, 2007). Exposure to stress or administration of a previously abused drug enhances the vulnerability to the reinstatement of drug seeking (De Wit & Stewart, 1981). Although the high rate of compulsive relapse to drugs during periods of abstinence is a primary clinical problem in treating drug abuse, the mechanisms underlying stress and drug priming-induced reinstatement of reward seeking have remained unclear.

The reinstatement Conditioned Place Preference (CPP) model has been widely used in many laboratories as an animal model of relapse to drug seeking (See, Fuchs, Ledford, & Mclaghlin, 2003; Shaham, Shalev, Lu, De Wit, & Stewart; 2003) that induced by stress, drug priming, or drug-associated cues after an extinction period (Parker & Mcdonald, 2000; Lu et al., 2002; Sadeghzadeh, Babapour, & Haghparast; 2015). Recently, growing evidence has shown that orexins are associated with numerous elements of addiction such as reward-related and drug-seeking behaviors (Harris, Wimmer, & Aston-Jones, 2005; Ebrahimian et al., 2016). Two receptors mediate effects of orexins: Orexin-1 (OX1R) and Orexin-2 (OX2R) (Sakurai et al., 1998). Orexin-containing neurons originate exclusively from the hypothalamus and directly send their projections throughout the central nervous system (De Lecea et al., 1998).

Several orexin projections associated with behavioral responses of drug abuse include the Nucleus Accumbens (NAc) and Ventral Tegmental Area (VTA) (Fadel & Deutch, 2002). All addictive drugs exert activatory effects on the dopaminergic neurons in the VTA that project into the NAc (Volkow, Wang, Tomasi, & Baler, 2013). Dopaminergic projections from the VTA to regions such as the NAc serve essential roles in mediating reinstatement of drug-seeking behaviors (Bossert, Poles, Wihbey, Koya, & Shaham, 2007).

Multiple lines of evidence have shown that orexin transmission play a key role in expression, extinction, and reinstatement of drug (Harris, Wimmer, & Aston-Jones, 2005; Smith, See, & Aston Jones, 2009; Guo et al., 2016; Sadeghzadeh, Namvar, Naghavi, & Haghparast; 2016).It is now well established that orexin signaling at the OX1R within VTA is necessary for the reinstatement of the drug (Wang, You, & Wise; 2009). Moreover, it has been reported that OX1R in VTA is involved in cue-induced cocaine-seeking behavior (James et al., 2011) and orexin signaling in Dentate Gyrus (DG) plays a crucial role in drug priming-induced reinstatement and a trivial role in Forced Swim Stress (FSS)-induced reinstatement (Ebrahimian et al., 2016). While the intracerebroventricular infusion of an OX1R antagonist suppresses footshock-induced reinstatement of cocaine seeking (Boutrel et al., 2005), intra-VTA administration of an OX1R antagonist does not prevent foot-shock-induced reinstatement (Wang, You, & Wise, 2009). It remains to be determined whether OX1R signaling within the VTA serves a role in mediating FSS- and morphine priming-induced reinstatement in a CPP paradigm.

In the present study, we tested whether the infusion of the OX1R antagonist, SB334867, into VTA modifies the relapse of morphine-seeking elicited by FSS and morphine priming previously associated with morphine availability.

## 2. Methods

### 2.1. Animal model

The experiments were performed on 88 adult male albino Wistar rats (200–280 g weight obtained from Pasteur Institute, Tehran, Iran) which were housed on a 12:12 h light/dark cycle at a temperature controlled room with free access to food and water. All methods used complied with guidelines for Care and Use of Laboratory Animals (National Institutes of Health Publication No. 80-23, revised 1996) and were approved by the Research and Ethics Committee of Shahid Beheshti University of Medical Sciences, Tehran, Iran.

### 2.2. Stereotaxic operation

The rats were mounted on a stereotaxic apparatus (Stoelting, USA) under ketamine 10% (100 mg/kg) and xylazine 2% (10 mg/kg) anesthesia. Then, using a stereotaxic apparatus, two guide cannulas were implanted 1 mm above the VTA (AP=4.8±0.15 mm caudal to bregma, Lat =±0.8 mm and DV=8.3 mm). These coordinates are in accordance with the rat brain atlas of Paxinos and Watson (Paxinos, Watson, 2004). The guide cannulas were secured in place using screws anchored to the skull and dental acrylic cement. After the operation, the animals were allowed to recover for 5–7 days.

### 2.3 Drugs

The drugs used in the present study were morphine sulfate (Temad, Iran) and SB334867 as a selective OX1R antagonist (Tocris Bioscience, Bristol, The United Kingdom). Morphine sulfate was dissolved in saline and SB334867 was dissolved in dimethyl sulfoxide (DMSO; Sigma-Aldrich, Germany). The control animals received either saline or 12% DMSO or both as the vehicle.

### 2.4. Microinjection Procedure

Microinjections were performed by attaching an injection needle (30-gauge injector cannula) to a Polyethylene tubing (PE-20). The free end of the tubing was attached to a 1-μL Hamilton syringe. An appropriate amount of injection was drawn (VTA: 0.3 μL/rat) into the tubing, and was infused over 60 second while the animal roamed freely.

### 2.5. Conditioning place preference paradigm

#### 2.5.1. Apparatus

The apparatus contains three-compartment conditioning boxes. The third one of which is considered as a start box, varying in size (30×15×40 cm) and usually serves as a connection between the other two equal-sized compartments (30×30×40 cm) and is separated by a removable Plexiglas wall. Conditioning took place in one of two compartments, which differed in pattern and texture. One compartment had white backgrounds with black vertical stripes and a smooth floor. Another compartment had black horizontal stripes with a rough floor. Before the conditioning session, the animals displayed no baseline preference for either of these two compartments.

#### 2.5.2. Conditioning place preference protocol

The Conditioned Place Preference (CPP) involves three phases: pre-conditioning, conditioning, and post-conditioning.

Pre-conditioning phase: In the pre-conditioning procedure (day 1), through the removal of the guillotine door, the animal was allowed to access all compartments freely. It was conducted to determine the baseline chamber preference and consisted of one 10-minute trial for each rat. Each animal displacement was recorded.

Conditioning phase: One day after the pre-conditioning session, the conditioning phase started consisting of six 30-min sessions (three saline and three drug pairing) in a 3-day schedule (the second day to the fourth day). These sessions were carried out twice each day, and the animals were restricted for 30 min after the first injection to one side of the two-sided compartment, returned to their home cages for 6 h, and then subjected to another 30-min conditioning trial. On each day, separate groups of animals received a conditioning session, one with drug and another with saline. The treatment compartment and order of presentation of drug/saline were counterbalanced for either group. Based on our recent studies (Haghparast et al., 2013; Alizamini, Farzinpour, Ezzatpanah, & Haghparast, 2017), 5 mg/kg Subcutaneous (SC) morphine was determined as the effective dose for the current experiments.

Post-conditioning phase: Each rat experienced a single CPP assessment session 24 h after the last conditioning session and done in a drug-free state. In this session, the rats were tested only once, having access to both compartments. The time spent and the distance traveled in each compartment for each rat during a 10-min period were recorded by a 3CCD camera (Panasonic Inc., Japan) and analyzed using the EthoVision software (XT, Version 7), a video tracking system for automation of behavioral experiments (Noldus Information Technology, the Netherlands). The conditioning score, as a preference index, was calculated as the time spent in the drug-paired compartment minus the time spent in the saline-paired compartment (Haghparast, et al., 2013). The total distance traveled by each animal was also documented in both control and experimental groups.

### 2.6. Extinction and reinstatement

**Figure 1.**
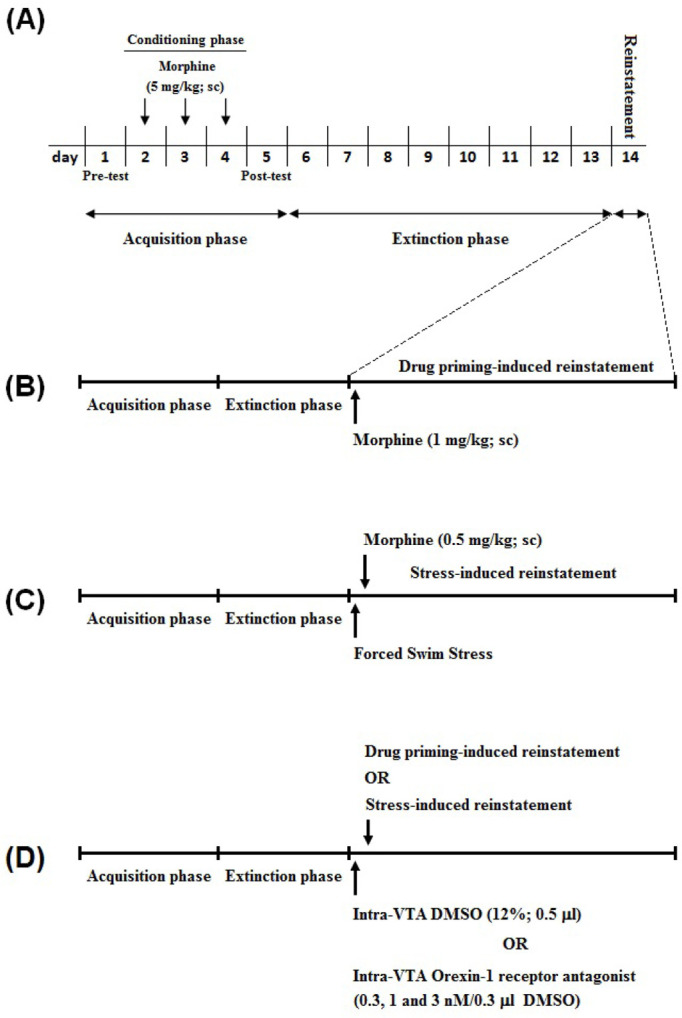
Experimental protocols for the FSS- and drug priming-induced reinstatement of morphine A. In the pre-conditioning phase (day 1) time spent in each compartment was recorded for all groups, and animals that did not show any preference for each compartment were included in this study. Following 3 days of morphine conditioning phase and a daily injection of morphine (5 mg/kg; SC), the CPP Test was performed on day 5 (post-conditioning test), CPP score was considered as the time spent in the drug-paired compartment minus the time spent in the saline-paired compartment; B. In this set of experiments for investigating the effect of morphine priming on reinstatement of morphine, under extinction condition, the rats were given “off” period (24 h), then the animals were placed in the CPP box and tested for place preference (CPP test) along with the injection of the ineffective dose of morphine (1 mg/kg); C. In another set of experiments for investigating the effect of FSS on the reinstatement of morphine, 24 h after the last day of extinction period, the animals were exposed to FSS and placed in the CPP box and tested for Place Preference (CPP test) 10 min later; D. The effect of bilateral injections of DMSO 12% as the vehicle or different doses of SB334867 as an orexin-1 receptor antagonist in the VTA on drug priming- and FSS-induced reinstatement of morphine were examined. CPP: Conditioned Place Preference; FSS: Forced Swim Stress; VTA: Ventral Tegmental Area.

After the conditioning procedure, the animals were located in the CPP apparatus (on days 6–13) in a drug-free state (extinction period) and CPP scores were calculated every day (Figure 1A). Extinction was accomplished when there was a lack of significant differences in CPP scores between 2 consecutive days in the extinction period and the pre-conditioning day (Sadeghzadeh et al., 2015; Ebrahimian et al., 2016). After a 24-h “off” period following the attainment of extinction criterion, the Reinstatement-test was performed by injection of a morphine priming (ineffective dose; 1 mg/kg, SC) or FSS (Figure 1B and C) before locating animals in the CPP apparatus with free access to all compartments. The duration of time consumed in each chamber was recorded for calculation of CPP score (Sadeghzadeh et al., 2015; Ebrahimian et al., 2016).

### 2.7. Forced swim stress

During this procedure, the rats were exposed on a 6-min forced to swim in a plastic cylindrical tank measuring 50 cm length ×30 cm width, that was filled with clean water (23°C–27°C) with a height of 30 cm (Ebrahimianet al., 2016). After the trial, the rats were dried with towels and returned to their home cages for at least 10 min before Reinstatement-test.

### 2.8. Experimental design

#### 2.8.1. The effect of intra-VTA injection of SB334867, on the forced swim stress-induced reinstatement of Morphine

Five groups of rats were used in this set of tests. There were two control groups; in non-FSS (NFSs) control group, the vehicle was infused into the VTA (0.3 μL 12% DMSO per side), and in another control group, the vehicle was infused into the VTA (0.3 μL 12% DMSO per side) 5 min before FSS. In the other three groups of rats, different bilateral doses of SB334867 (0.3, 1, and 3 nM/0.3 μL 12% DMSO per side) were administered into the VTA, 5 min before FSS. Ten minutes after FSS, all three groups of rats were located into the CPP boxes for the Reinstatement-test and permitted to move freely for 10 min. The CPP score and the distance traveled were documented (Figure 1D).

#### 2.8.2. The effect of intra-VTA injection of SB334867, on the Morphine priming-induced reinstatement

In this set of experiments, five groups of animals were used. In one control group, intra-VTA vehicle (0.3 μL 12% DMSO per side) and subcutaneous saline were injected. In another control group, intra-VTA vehicle (0.3μl 12% DMSO per side) was administered 5 min before the Reinstatement-test, and a subcutaneous morphine priming (1 mg/kg) was injected immediately before rats placed into the CPP chamber for the Reinstatement-test. The other three groups of rats received different bilateral doses of SB334867 (0.3, 1, and 3 nM/0.3 μL 12% DMSO per side) in the VTA, 5 min before the reinstatement induced by a morphine priming (1 mg/kg; SC). The animals were located into the CPP boxes for 10 min and permitted to move freely, and the CPP score and distance traveled were documented (Figure 1D).

### 2.9. Histological verification

At the end of the experiments, the rats were deeply anesthetized with ketamine and xylazine and perfused transcardially with a 0.9% saline solution followed by a solution of 10% formalin. Their brains were removed and stored in 10% formalin. The neuroanatomical location of cannulas tips were confirmed using the rat brain atlas (Paxinos & Watson, 2004). The data reported here are only from animals in which the placements of cannula tips were histologically verified.

### 2.10. Statistical Analysis

The obtained data are presented as Mean±SD for each experimental group. To compare the conditioning scores or locomotor activity obtained in all control and experimental groups, 1-way Analysis of Variance (ANOVA) followed by post hoc analysis (Newman-Keuls) was used, as per appropriate. The statistical significance was set at P<0.05.

## 3. Results

### 3.1. The effect of intra-VTA administration of SB334867, on the forced swim stress-induced reinstatement of Morphine

**Figure 2.**
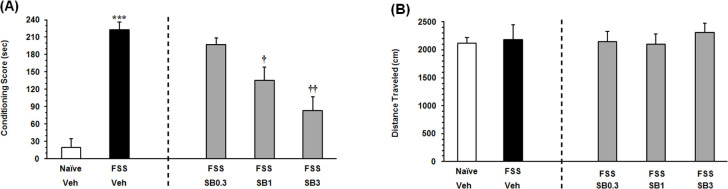
The key findings of present study specified that 1. The FSS-induced reinstatement was suppressed by the blockade of OX1R within VTA; 2. Blockade of the OX1R in the VTA significantly prevented reinstatement induced by morphine priming.

The Independent samples t-test result (t_11_= 9.967, P<0.001) revealed that the animals exposed to the FSS, consumed more time on the morphine-paired side in comparison with NFSS group (Figure 2A on the left panel). To evaluate the effect of the OX1R antagonist on the FSS-induced reinstatement, the animals received three doses of SB334867 microinjection into the VTA. The 1-way ANOVA followed by the Dunnett’s Multiple Comparison test result (F_3, 25_=10.49, P<0.0002) indicated a significant difference between the FSS group and the animals that received two higher doses of SB334867 (1 and 3 nM/0.3μL 12% DMSO per side; Figure 2A, right panel) in the reinstatement CPP score. It showed intra-VTA administration of SB334867 could modify FSS-induced reinstatement. Statistical analysis indicated that FSS and intra-VTA administration of the OX1R antagonist did not affect the locomotor activity (F_4,32_=0.2091, P=0.9312) Figure 2B.

### 3.2. The effect of intra-VTA administration of SB334867, on the Morphine priming-induced reinstatement

**Figure 3.**
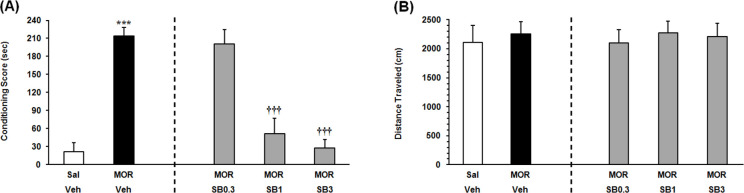
Effects of microinjections of the vehicle (Veh), and different doses of SB334867 into the VTA on morphine priming-induced reinstatement A. Left panel, the animals received ineffective doses of morphine (1 mg/kg) and saline (1 ml/kg), Right panel: the animals received ineffective doses of morphine (1 mg/kg) and different doses of SB334867 within VTA. Intra-VTA administration of SB334867 could block morphine priming-induced reinstatement; B. Mean locomotor activity of all groups. All data are expressed as Mean±SD for six to seven rats; *** P<0.001. The morphine-control group (Mor-Veh) compared saline-control group (Sal-Veh); *** P<0.001.The SB334867-treated group compared to the morphine-control group (Mor-Veh).

As indicated in Figure 3A on the left panel, the Independent samples t-test result (t_9_=9.506, P<0.001) presented that the rats that received an ineffective dose of morphine, consumed more time on the morphine-paired side in comparison with the saline-vehicle control group. It showed that an ineffective dose of morphine would induce significant reinstatement. To examine the effect of intra-VTA OX1R antagonist on the morphine priming-induced reinstatement, the animals received three doses of SB334867 microinjection into the VTA. The 1-way ANOVA followed by the Dunnett’s Multiple Comparison test result (F_3,22_=22.49, P<0.0001) indicated a significant difference between the morphine priming group which received vehicle and morphine priming groups which received the two higher doses of SB334867 in the reinstatement of CPP (1 and 3 nM/0.3 μL 12% DMSO per side) (Figure 3A, right panel). It showed that intra-VTA administration of SB334867 could block morphine priming-induced reinstatement. The 1-way ANOVA followed by Newman–Keuls’ post hoc analysis (F_4, 28_=0.1187, P=0.9745) indicated that priming dose of morphine and intra-VTA administration of the OX1R antagonist did not affect the locomotor activity (Figure 3B).

## 4. Discussion

The present study aimed to investigate the role of intra-VTA OX1R in the reinstatement of morphine-seeking behavior induced by FSS and drug priming in rats. The key findings of present study specified that 1. the FSS-induced reinstatement was suppressed by the blockade of OX1R within VTA; 2. blockade of the OX1R in the VTA significantly prevented reinstatement induced by morphine priming. The intra-VTA infusion of none of these drugs altered locomotor activities in comparison with the vehicle-control groups.

In agreement with previous studies, we found that the VTA appears to be a critical part of the neural system associated with all drugs abuse (Winder, Egli Schramm, & Matthews, 2002). Both orexin receptor subtypes (OX1R and OX2R) are expressed at high levels in the VTA (Narita et al., 2006). It has been reported that stimulation of the orexinergic neuron in the VTA activates mesolimbic dopaminergic neurons directly (Narita et al., 2006). These studies highlight the imperative behavioral role of VTA regulated by orexin inputs.

The main finding in the present experiments is that bilateral intra-VTA administration of different doses of the OX1R antagonist reduces acute FSS-induced reinstatement. Our results, consistent with the results of Boutrel et al. (2005), revealed the importance of orexin signaling in drug-seeking elicited by stress (Boutrel et al., 2005). However, there is some inconsistency in studies, as another major study demonstrates that the intra-VTA infusion of an OX1R antagonist does not significantly affect drug-seeking behavior induced by footshock (Wang, You, & Wise, 2009).

The plausible reason for this discrepancy is that the neurocircuitry underlying FSS- and footshock-induced reinstatement of drug seeking may not be identical. It seems that footshock only stimulates orexin neurons in the Dorsomedial Hhypothalamus (DMH) and Perifornical Area (PFA) but not in the Lateral Hypothalamus (LH) (Harris, Wimmer, & Aston-Jones, 2005). Growing evidence has supported the hypothesis that orexin neurons in PFA and DMH are involved in arousal whereas those in LH are involved in the reward system (Estabrooke et al., 2001; Harris, Wimmer, & Aston-Jones, 2005).

We propose that behavior responses contributing to FSS but not footshock regulate LH orexin-expressing neurons associated with the re ward system. Also, our results are almost intriguing with our previous findings, which indicated an indirect role of OX1R within DG in the reinstatement of FSS-induced morphine seeking (Ebrahimian et al., 2016). This discrepancy cannot be elucidated by a dose effect, because the highest dose used in our previous study was three times higher than that used in the current study. It appears that this inconsistency may primarily result from the injection site centered to DG versus the VTA injection site used in our study. These data support the idea that OX1Rs within the VTA but not DG may serve a direct and main role in the FSS-induced reinstatement.

Another conclusion might be drawn from this study is the prominent role of OX1R in morphine priming-induced reinstatement. Multiple lines of evidence have been demonstrated that activation of LH orexin neurons or intra-VTA administration of the orexin-A peptide reinstated morphine-seeking as measured by the CPP (Harris, Wimmer, & Aston-Jones, 2005). In another major study, Wang et al. investigated orexin-A (non-selective agonist for OX1R and OX2R) but not orexin-B (selective agonist for OX2R) provoked cocaine-seeking and triggered release of VTA glutamate and dopamine, supporting the idea that orexin-neurotransmission at OX1R was involved mainly in reward-seeking (Wang, You, & Wise, 2009). A wealth of evidence also indicates intra-VTA and systemic administration of OX1R antagonist reduce cue-induced reinstatement of the drug (Smith, See, & Aston-Jones, 2009; James et al., 2011). Moreover, blockade of the OX1R within DG inhibits the morphine priming-induced reinstatement of CPP. These results in line with the current data strongly support the idea that activation of the orexin-containing neurons in the VTA leads to reward seeking provoked by morphine priming.

The mechanism of orexin signaling during reinstatement of morphine seeking is unclear. Converging evidence reveals that the orexinergic system may regulate the activation of the mesolimbic dopamine system (Narita et al., 2006). Morphine is known to activate mesolimbic dopamine neurons mostly by preventing GABAergic interneuron activity via the stimulation of μ-opioid receptors. However, it may be possible that the activation of mesolimbic dopamine neurons by morphine is, at least in part, regulated by the activation of orexin-containing neurons via μ-opioid receptors on the orexin neurons in the LH, which send their projections to the VTA (Narita et al., 2003; Narita et al., 2006). However, additional behavioral, electrophysiological, and molecular studies are essential for clarifying the hypothesis of the neural mechanism of orexin in reward seeking and other interactions between orexinergic and reward systems within the VTA. The current study demonstrates the role of orexin signaling via OX1R in morphine seeking; nevertheless, the effect of OX2R antagonist on the reinstatement of morphine seeking is not so clear, and it is worth to be potential research topics for future studies.

Overall, we found that the activation of orexin transmission at OX1R in the VTA is directly implicated in the reward-seeking behavior induced by stress and morphine. We conclude that the orexin system may serve an important functional role in drug seeking and vulnerability to relapse. The orexin system may, therefore, be a target for preventing relapse during prolonged abstinence.
